# Scientific Items

**Published:** 1886-11-20

**Authors:** 


### SCIENTIFIC ITEMS.

**Recent** investigations in Germany appear to have established the fact that the amount of damage done by lightning in that country is rapidly increasing from year to year. In 1869 the well-known meteorologist, Professor von Bezold, published the results of an investigation he had carried out, based upon the books of the Bavarian Fire Insurance, in which about 30 per cent. of all existing buildings are insured. In these books accurate account would be kept of all the cases in which lightning had affected any of the insured buildings. The first results of this investigation showed that there was a regular increase going on in the number of instances of lightning striking the buildings, reckoned as percentage on the buildings, of course; and a later publication in 1884, giving a continuation and more exact and complete examination of the figures obtained, showed the result to be, that during the fifty years from 1833 to 1882 the percentage of insured buildings struck by lightning in Bavaria had increased at least threefold. The publication of these facts led to similar investigations in Saxony and other parts of Germany, with very similar results. It would be interesting to know whether there has been a similar increase in damage from lightning in this country during recent years.—*Popular Science News.*

**Development of the Human Body.**—During the International Medical Conference held at Copenhagen, the Rev. Malling Hansen, Principal of the Danish Institution for the Deaf and Dumb, presented a paper which attracted considerable interest. It gave the daily results of weighing and measuring the 130 pupils (72 boys and 58 girls) of the institution during a period of three years. The facts demonstrated by these statistics were quite a surprise to the medical people in attendance. Since this preliminary notice, given in the summer of 1884, Mr. Hansen has continued his observations, and now believes himself able to furnish some outline of bodily development. Each child was weighed four times a day—in the morning, before dinner, after dinner, and in the evening; and measured once. These daily records show that, contrary to general opinion, the increase in weight and height of the human body during the years of growth does not progress evenly throughout the year. Three distinct periods were observed, and smaller variations were noticeable within these divisions. In bulk, the period of maximum increase extends from August to December. A period of equipoise then succeeds until the middle of April, and the following minimum period completes the year. The lasting increase in weight occurs during the first period; the period of equipoise adds one fourth of that increase, but this is almost entirely spent during the last period.

The increase in height shows a similar division into periods, but in a reverse order. In September and October, a child grows only a fifth of what it did in June and July. Thus in the autumn and early winter a child increases in weight, while the height remains stationary. In the early summer, on the contrary, the weight changes but little, while the vital force and nourishment are directed toward an increase in height. This periodicity in the development of the body marks a strong similarity to plant development, and it is quite probable that further investigations will show another likeness in the fact that these results are good only for the latitude in which they were obtained. In a climate less variable than that of Denmark, it is highly probable that the periods would be less marked, and in an even temperature would cease to be distinguished.—*Scientific American.*

**The Southern Hemisphere** seems of late years to be unusually afflicted with earthquakes and volcanic eruptions. The eruption of two summers ago in the Javanese archipelago, when an entire island was blown to pieces and partially submerged, is still fresh in our memory; and recent reports from New Zeland state that a chain of extinct volcanoes has suddenly been aroused to action, and that a section of country twenty-five miles wide and over a hundred miles long was in a single night converted into a "land of fire." Whole villages have been covered with volcanic ashes, and destroyed; and the loss of life has been very great. Compared with this great explosion, the Javanese eruption becomes comparatively insignificant, and the ancient eruption of Vesuvius which destroyed Herculaneum and Pompeii hardly worthy of mention. It will be interesting to note whether the great eruption will be followed by the same remarkable after-glows, or "red sunsets," which occurred in the fall of 1884, and were attributed to the immense masses of fine dust thrown into the air during the eruption of the preceding summer.—*P. S. News.*

**Comparative Longevity of the Sexes.**—The Hebrew women are the longest-lived, and the colored men the shortest. It appears from the gathered statistics of the world, that women have a greater tenacity of life than men. Nature worships the female in all its varieties. Among insects the male perishes at a relatively earlier period. In plants the seminate blossoms die earliest, and are produced in the weaker limbs. Female quadrupeds have more endurance than males. In the human race, despite the intellectual and physical strength of man, woman endures longest, and will bear pain to which the strong man succumbs. Zymotic diseases are more fatal to males, and more male children die than female. Deverga asserts that the proportion dying suddenly is about 100 women to 700 men. 1,080 men in the United States, in 1870, committed suicide, to 285 women. Intemperance, apoplexy, gout, hydrocephalus, affections of the heart or liver, scrofula, paralysis, are far more fatal to males than females. Pulmonary consumption, on the other hand, is more deadly to the latter. Females in cities are more prone to consumption than in the country. All old countries not disturbed by emigration have a majority of females in the population. In the royal families the statistics show more daughters than sons. The married state is favorable to prolongation of life among women. Dr. Hough remarks that there are from 1 to 6 per cent. more males born than females, yet there is an excess of more than 6 per cent. of females in the living population.—*Ibid.*

